# Predictive factors for intrauterine growth restriction

**Published:** 2014-06-25

**Authors:** AR Albu, AF Anca, VV Horhoianu, IA Horhoianu

**Affiliations:** “Carol Davila" University of Medicine and Pharmacy, Bucharest, Romania University Emergency Hospital Bucharest Obstetrics and Gynecology Unit

**Keywords:** intrauterine growth restriction, serum markers, PAPP-A, uterine artery Doppler, high-risk pregnancy

## Abstract

Abstract

Reduced fetal growth is seen in about 10% of the pregnancies but only a minority has a pathological background and is known as intrauterine growth restriction or fetal growth restriction (IUGR / FGR). Increased fetal and neonatal mortality and morbidity as well as adult pathologic conditions are often associated to IUGR. Risk factors for IUGR are easy to assess but have poor predictive value. For the diagnostic purpose, biochemical serum markers, ultrasound and Doppler study of uterine and spiral arteries, placental volume and vascularization, first trimester growth pattern are object of assessment today. Modern evaluations propose combined algorithms using these strategies, all with the goal of a better prediction of risk pregnancies.

Abbreviations: SGA = small for gestational age; IUGR = intrauterine growth restriction; FGR = fetal growth restriction; IUFD = intrauterine fetal demise; HIV = human immunodeficiency virus; PAPP-A = pregnancy associated plasmatic protein A; β-hCG = beta human chorionic gonadotropin; MoM = multiple of median; ADAM-12 = A-disintegrin and metalloprotease 12; PP-13 = placental protein 13; VEGF = vascular endothelial growth factor; PlGF = placental growth factor; sFlt-1 = soluble fms-like tyrosine kinase-1; UAD = uterine arteries Doppler ultrasound; RI = resistence index; PI = pulsatility index; VOCAL = Virtual Organ Computer–Aided Analysis software; VI = vascularization index; FI = flow index; VFI = vascularization flow index; PQ = placental quotient

## What is intrauterine growth restriction?

 Intrauterine growth restriction is defined as the pathologic inhibition of intrauterine fetal growth and the failure of the fetus to achieve its growth potential [**1**]. Considered by the American College of Obstetricians and Gynecologists “the most common and complex problem in modern obstetrics", fetal growth restriction still needs clear criteria because intrauterine fetal growth is not defined by clear parameters but mainly estimated on the basis of multiple factors. Research in this field is trying to find predictive parameters, with the goal of reaching an early diagnosis, which would lead to a better management of the condition [**2**].

Impaired fetal growth is seen in about 10% of pregnancies [**3**]. While the majority of such pregnancies will have a physiologically normal fetus that is simply small for gestational age (SGA), pathological fetal growth-restriction is a different category and the ability to differentiate such a condition from SGA is limited. A healthy fetus with estimated weight or birth weight below the 10th percentile according to population standards is commonly defined as SGA [**4,5**]. Pathological SGA is known as intrauterine growth restriction (IUGR) or fetal growth restriction (FGR). Fetal development is evaluated through comparison between estimated fetal weight or birth weight and references or standards for different gestational ages [**6**].

 Why detecting intrauterine growth restriction?

 Fetal growth is known to be an important predictor of pregnancy outcome and reflects the interaction between physiological and pathological factors influencing the fetus [**7-9**]. IUGR is associated with increased fetal and neonatal mortality and morbidity, being linked to immediate perinatal adverse events (prematurity, cerebral palsy, intrauterine fetal death, neonatal death) and also to adult pathologic conditions (obesity, hypertension, type-2 diabetes) [**10-14**]. Former studies showed that cerebral palsy was found to be 4-6 times more frequent in newborns below the 10th percentile compared to those between the 25th and the 75th percentile [**15**]. A strong association between FGR and stillbirth was also confirmed [**16**]. From the moment FGR was recognized as a cause of perinatal death, almost 50% of stillbirth were identified as FGR [**17**], resulting in a decrease of unexplained stillbirth from 67-70% to 15% [**18**]. Therefore, fetal growth impairment identification triggers a 4-time reduction in neonatal complications and death [**14**]. 

 In about 75% of the cases, IUGR remains unrecognized until birth and the diagnosis comes retrospectively, whereas in low-risk pregnancy the detection rate is about 15% [**19**]. In addition, the investigation of possible IUGR/FGR has the objective of an early detection and appropriate management that could lower the percentage of stillbirths and other perinatal complications, hoping that the identification of low risk fetuses would decrease the number of unnecessary surveillance [**11**]. 

 IUGR: risk factors

 The risk factors for IUGR comprise a wide range of conditions and their assessment should be seriously taken into account, as they are easy to perform and are routinely used during pregnancy [**20,21**]. The main factors assessed in clinical practice include: maternal factors [socioeconomic status, weight (very low and also increased body mass index), smoking, use of recreational drugs, advanced maternal age, nulliparity, history of gestational hypertension, family history of IUGR or previous IUGR pregnancy, previous pregnancy with preeclampsia, IUFD, inherited or acquired trombophilia, anemia, high altitude living, autoimmune disorders (phospholipid syndrome, lupus erythematosus), antepartum diabetes mellitus, cronic diseases (chronic pulmonary disease, cyanotic heart disease)], fetal factors [multiple gestation, congenital infections (Cytomegalovirus, Syphillis, Rubella, Varicella, Toxoplasmosis, Tuberculosis, HIV, Malaria), aneuplodies (trisomy 13, 18, 21, triploidy), genetic syndromes], adnexal factors [uterine malformations, subchorionic haematoma, extensive villous infarction, marginal or velamentous cord insertion, placental mosaicism] [**22**].

 A positive history for risk factors of IUGR can raise the problem of an increased surveillance with the specific goal of an early detection of growth insufficiency [**23**]. Further diagnostic tests could have a better relevance in a selected high-risk population [**24**]. However, these clinical factors have shown a different impact for each individual case and their relevance, as effective screening tools have not been proved yet [**25**]. 

 As presented above, IUGR/FGR is linked to serious pregnancy complications and early assessment of the condition is a main topic of research today. Furthermore, preventive measures like administration of low dose aspirin showed efficacy in IUGR treatment if administered before the 16th week of gestation [**26**]. For this purpose, biochemical serum markers, Doppler study of uterine arteries, placental volume and vascularization as well as first trimester growth pattern, were the object of study for IUGR/FGR assessment.

 Serum markers linked to IUGR

 The placentation process starts with the migration of trophoblastic cells that invade the walls of spiral arteries and transform them from small caliber high resistant vessels into wide caliber low resistant vessels that deliver blood at low pressure to the intervillous space. Then, the utero-placental circulation develops in two stages: the first stage (until the 10th week of gestation) consists in endovascular plugging of the spiral arteries by trophoblastic cells, subsequently followed by invasion and destruction of the intradecidual spiral arteries; the second stage (between 14-16 weeks of gestation) consists in the invasion of the inner miometrial part of the spiral arteries [**27**]. The impaired spiral artery transformation is leading to weak development of the utero-placental circulation and is implied in the pathology of preeclampsia and IUGR [**21**].

 Maternal serum analytes were first studied with the aim of screening for aneuploidies during the first or second trimester of pregnancy and their use was then further extended in many studies to evaluate their utility as markers for impaired placentation. The trophoblastic invasion failure is thought to be responsible of the changes in the concentration of serum placental products [**21**]. Several serum analytes have been studied in hope of finding a relevant marker linked to IUGR, but none of them proved to be sufficiently accurate to be used in routine clinical practice as single predictive marker. The combined approach using clinical data, serum markers, biophysical parameters (ultrasound parameters, arterial blood pressure) showed increased predictive relevance [**28**].

 Pregnancy associated plasma protein A (PAPP-A), an Insulin–like Growth Factor Binding Protein Protease whose levels depend on placental volume and function, was assessed in several studies with congruent results. In 2000, Ong et al. evaluated 5584 singleton pregnancies at 10-14 weeks of gestation and measured maternal serum free beta human chorionic gonadotropin (β-hCG) and PAPP-A, concluding that low levels of maternal serum PAPP-A or β-hCG were associated with subsequent development of pregnancy complications [**29**]. Furthermore, in the group of 4390 women with singleton pregnancy evaluated by Spencer et al. in 2005 the maternal low levels of serum PAPP-A at 11-13 weeks of gestation resulted significantly associated to adverse pregnancy outcomes [**30**]. Low levels of PAPP-A were mentioned as predictors of IUGR also by Goetzinger et al. in 2009 and Poon et al. in 2009 [31,32]. The sensitivity of detecting IUGR for a first trimester PAPP-A level below the 5th percentile ranges only between 8% and 33% and PAPP-A as single marker is an insufficient screening tool for IUGR [**21,33**]. 

 Dugoff et al. evaluated 34271 pregnancies at 11-14 weeks of gestation and proved that maternal serum free β-hCG was mildly reduced in pregnancies that subsequently developed IUGR [**34**]. Later, Gotzinger et al. showed that high levels of β-hCG were linked to SGA fetuses [**31**]. Overall, the predictivness of free β-hCG is unsatisfactory today [**21,30,25**].

 In 2008, in the name of the Genetics Committee of the Society of Obstetricians and Gynaecologists of Canada, Gagnon et al. reviewed the obstetrical outcomes associated with abnormal levels of single or multiple maternal serum markers used in the screening for aneuploidy. PAPP-A, alphafetoprotein, β-hCG, estriol, unconjugated estriol, inhibin-A were the analytes assessed and among the conclusions an unexplained low PAPP-A (< 0.4 MoM) and/or a low hCG (< 0.5 MoM) in first trimester were associated with an increased frequency of adverse obstetrical outcomes [**36**].

 As in the first trimester biochemical screening starts as early as 8wa, the detection of a low level of hyperglycosylated human chorionic gonadotrophin correlated with a low PAPP-A and resulted a marker of impaired placentation, showing 90% specificity and 69% sensitivity when adjusted for mean arterial pressure and parity [**37**].

The A-disintegrin and metalloprotease 12 (ADAM-12) is an Insulin–like Growth Factor Binding Protein Protease produced by the placenta, and results into another potential marker of preeclampsia and FGR when tested in the first trimester, but with a low predictive value and a detection rate ranging between 7,16% and 20% [38-40].

 Placental Protein 13 (PP-13) is a peptide involved in placental implantation and vascular invasion and remodeling [**41**]. In 2007, Chaftez et al. showed that low levels of first trimester PP-13 were associated with preterm birth in women with IUGR [**42**]. In 2008, Spencer et al. found poor association between PP-13 and pregnancy outcome like IUGR [**43**]. In 2008, using the integrated assessment of PP-13, PAPP-A and uterine artery Doppler at 11-13 weeks of gestation, Poon et al. determined a 20% detection rate of FGR at a false positive rate of 5% [**44**].

 Angiogenic factors implied in placentation like Vascular Endothelial Growth Factor (VEGF) and Placental Growth Factor (PlGF) were also evaluated in many studies [**45**]. Soluble fms–like tyrosine kinase-1(sFlt-1) on the other hand, inhibits the effects of VEGF and PlGF by blocking their interaction with the specific receptors. The imbalance between the anti-angiogenic and angiogenic factors seems to be linked of preeclampsia and IUGR [**45**]. The recent meta-analysis performed by Conde-Agudela et al. in 2013 including 53 studies and 39974 women evaluated 37 new biomarkers among which: Angiogenesis-related biomarkers [Placental Growth Factor, Soluble fms-like tyrosine kinase-1, Soluble endoglin, Vascular endothelial growth factor], Endothelial function/oxidative stress-related biomarkers [Homocysteine, Leptin, Asymmetric dimethyl-arginine, Soluble Vascular Cell Adhesion Molecule-1, Interferon-c, C-reactive Protein, Folate]; Placental proteins/hormone-related biomarkers [Insulin-like Growth Factor Binding Protein-1 and -3, ADAM-12, PP-13, Activin A, Placental Growth Hormone] and other markers like metabolomics and genetic biomarkers. Overall, none of the 37 novel biomarkers evaluated showed a significant accuracy for predicting IUGR [**45**].

 Ultrasound markers

 The Doppler ultrasound sampling of uterine arteries (UAD) is a useful non-invasive method for the assessment of the interaction between fetal and maternal hemodynamic compartment. Starting from the early studies of Campbell et al. and Trudinger et al. in the 1980s, this procedure has been reported in numerous following surveys and showed to be a promising technique for the prediction of the IUGR risk [46-48]. In case of an incomplete trophoblastic invasion of the spiral arteries, the maternal compartment failsed to transform from a high resistance to a low resistance flow district and this impairment is associated with the development of preeclampsia and IUGR. In these cases, a Resistence Index (RI) or a Pulsatility Index (PI) above the 90th-95th percentile and/or the presence of unilateral or bilateral notching is associated with a value of the RI >0,58 or a value of the PI >1,45 [**21**].

 The early studies that assessed UAD were performed during the second trimester of gestation, between weeks 18-23 [49-54]. Although an increased resistance in the uterine arteries is associated with poor obstetric outcome like preeclampsia, IUGR or prematurity, the positive predictive value was found to be only 15% for IUGR [**49**]. Therefore, in 1994 North et al. and in 1998 Irion et al. stated that UAD was not a reliable screening test for nulliparous women [**50,51**]. Further studies performed in the 2000s have evaluated UAD between 10-14 weeks of gestation finding low positive predictive values for IUGR and a slightly better prediction value for preeclampsia with IUGR or IUGR alone with delivery in less than 32 weeks [**53,55**]. Most of the studies published to date, disregarding if performed during the first or second trimester, agree that UAD modifications are linked to IUGR but are not a reliable single predictive marker for defining a low risk category [**56**]. An interesting approach proposed by Gomez et al. in 2006 showed that the sequence of changes in the uterine flow between the first and second trimester correlates with subsequent appearance of IUGR and the highest risk is held by women with persistent low vascular indices [**54**]. 

Spiral arteries have been subjected to assessment during the first trimester. Mäkikallio et al. found that spiral arteries impedance decreased starting with the 5th week of gestation and uterine and arcuate artery lost their tonus starting with the 8th week [**57**]. Hung et al. evaluated color and spectral Doppler of the spiral arteries between 13-19 weeks and 20-25 weeks concluding that spiral artery Doppler assessment was not a sensitive tool for detection of IUGR in a low risk population [**58**]. Furthermore, in studies comparing normal outcome pregnancies versus complicated pregnancies, spiral arteries sampling failed to show any statistically significant predictive value, making spiral arteries Doppler study an insufficient method for screening of IUGR [**59**].

The vasculature of the placental villous tree and the placental size are related to the invasion of the spiral arteries and thus predictors of pregnancy complications like preeclampsia and IUGR. After 1994, when Rubin et al. described the use of Power Doppler ultrasound assessment, many researchers evaluated placental volume and placental vascularization by using Color or Power Doppler and 3D/4D scanning techniques [60,61].

 The introduction of Virtual Organ Computer–Aided Analysis software (VOCAL) proved useful in the quantification of blood vessel volume and of the flow in an acquired volume [**62**]. In a region of interest, three indices are mostly calculated and used: Vascularization Index (VI): the ratio between color vessels and total vessels (color and grey scale vessels), representing the percentage of blood vessels in a region of interest; the Flow Index (FI): the sum of a color vessel’s signal intensity divided by the number of color vessels, representing the amount of blood corpuscles in the vessels in a region of interest; the Vascularization Flow Index (VFI): the ratio between the sum of the color vessel’s signal intensity and the total tissue vessels, representing the total number of blood corpuscles in the total tissue volume. In 2004, Merce et al. showed that the 3D-Power Doppler study of the placental vascularization in normal pregnancies is reproducible and seems to be a useful tool in performing “a placental biopsy" [**63**]. In 2008, Guiot et al. used this technique in the assessment of the 3rd trimester pregnancies complicated with IUGR and compared them to normal pregnancies, correlating VI, VFI and FI values to Umbilical Artery Doppler abnormalities (elevated Systolic/Diastolic ratios and absent End Diastolic Velocity), and the results showed that modifications in vascularization indices could be detected earlier than Umbilical Artery Doppler abnormalities [**64**]. In 2009, Noguchi et al. performed “placental vascular sonobiopsies" starting with the second trimester, comparing normal and growth restricted pregnancies and proving the differences in vasculature [**65**]. Against these evidences, in vitro phantom studies performed by Raine-Fenning et al. and Schulten-Wijman et al. in 2008 questioned the technical and methodological aspects of this procedure, doubting the reproducibility¬¬¬¬ [**66,67**]. On the other side, the sheep model study conducted by Olivier Morel et al. in 2010 demonstrated a good correlation between true uterine blood flow and flow parameters, as well as a good reproducibility of the technique [**68**].

 In regard to placental volume assessment Rizzo et al. showed in 2009 that first trimester evaluation of placentas in a high risk group with low PAPP-A proved a good correlation between reduced placental volume and low fetus weight (<10th percentile) with Umbilical Artery Doppler modifications [**69**]. Another way of determining early fetal growth was proposed by Hafner et al. who compared Placental Quotient (PQ) [Placental Volume / Crown Rump Length] assessed during the first trimester with Uterine Artery Doppler at 22 weeks and concluded that PQ has low sensitivity in diagnosis of IUGR in low-risk population, comparable with that of Uterine Artery Doppler, but has the advantage of possible first trimester assessment [**70**]. In 2013, these authors refined their research showing that Placental Bed Vascularization Index assessed by 3D Power Doppler could be used for a quick and reliable first trimester assessment of severe pregnancy risks because it shows a 66,2% detection rate for severe pregnancy complications, higher than the 50% sensitivity of second trimester Uterine Artery Doppler [**71**]. 

 The multiparameter approach

 Investigators tried to improve the prediction of IUGR by combining Doppler indices with biochemical and clinical parameters due to the insufficient predictive value of each marker alone [**21**]. The combined approach reflects various pathological pathways: Doppler ultrasound of the uterine arteries shows inadequate invasion of the spiral arteries and impaired secretory placental function is reflected by the disturbances of biomarker levels [**72**]. Preeclampsia is associated with IUGR and models of prediction by combining ultrasound assessment of uterine arteries, biochemical markers and maternal history were published concerning the assessment of both conditions, but the need for the development of a model for prediction of SGA without preeclampsia emerged [**72-74**]. With the purpose of identifying SGA fetuses not related to preeclampsia, Karagiannis et al. used a model of prediction containing Uterine Artery Doppler Pulsatility Index at 11-13 weeks of gestation, Mean Arterial Pressure, maternal serum PAPP-A, free β-hCG, Placental Growth Factor, PP-13, ADAM-12 and Fetal Nuchal Translucency. Although the study did not distinguish between the constitutional and pathological SGA, it raised the problem of increased surveillance for the whole population at risk for SGA, improving perinatal outcome by individualized management in late-second trimester and third trimester. The study also proved a good detection rate for SGA of 73% for those requiring delivery before 37 weeks and of 46% for those requiring delivery after 37 weeks [**28**].

## Conclusions

Although it is a public health problem with around 10% incidence in the pregnancy population, FGR is still a matter of debate starting from the definition, the multifactorial origin, the low levels of prediction, the questionable references like customized or uncustomized growth charts, and the methods of diagnosis and management. The main trajectory of research is towards the first trimester diagnosis, as the optimal interval in which a prophylactic treatment would influence placentation ends in the 16th week [**26**]. A thorough history should be taken to all pregnant women at the first antenatal visit because risk factors determination results are easy and inexpensive. Different single approaches in the screening of this condition have been proposed but did not show sufficient sensitivity, therefore, combined models are proposed with the goal of a better predictive value. Large-scale prospective studies are required to prove the power of such integrated approaches in clinical practice.
